# Downregulation of Mcl-1 by Panobinostat Potentiates Proton Beam Therapy in Hepatocellular Carcinoma Cells

**DOI:** 10.3390/cells10030554

**Published:** 2021-03-04

**Authors:** Changhoon Choi, Ga Haeng Lee, Arang Son, Gyu Sang Yoo, Jeong Il Yu, Hee Chul Park

**Affiliations:** 1 Department of Radiation Oncology, Samsung Medical Center, Seoul 06351, Korea; chchoi93@gmail.com (C.C.); vitamin_milk@naver.com (G.H.L.); onlyshohow@naver.com (A.S.); gs.levin.yoo@samsung.com (G.S.Y.); jeongil.yu@samsung.com (J.I.Y.); 2 School of Medicine, Sungkyunkwan University, Seoul 06351, Korea

**Keywords:** proton therapy, hepatocellular carcinoma, panobinostat, Mcl-1

## Abstract

Epigenetic modulation by histone deacetylase (HDAC) inhibitors is an attractive anti-cancer strategy for diverse hematological and solid cancers. Herein, we explored the relative effectiveness of the pan-HDAC inhibitor panobinostat in combination with proton over X-ray irradiation in HCC cells. Clonogenic survival assays revealed that radiosensitization of Huh7 and Hep3B cells by panobinostat was more evident when combined with protons than X-rays. Panobinostat increased G2/M arrest and production of intracellular reactive oxygen species, which was further enhanced by proton irradiation. Immunofluorescence staining of γH2AX showed that panobinostat enhanced proton-induced DNA damage. Panobinostat dose-dependently decreased expression of an anti-apoptotic protein, Mcl-1, concomitant with increasing acetylation of histone H4. The combination of panobinostat with proton irradiation enhanced apoptotic cell death to a greater extent than that with X-ray irradiation. Depletion of Mcl-1 by RNA interference enhanced proton-induced apoptosis and proton radiosensitization, suggesting a potential role of Mcl-1 in determining proton sensitivity. Together, our findings suggest that panobinostat may be a promising combination agent for proton beam therapy in HCC treatment.

## 1. Introduction

Hepatocellular carcinoma (HCC) is the sixth most common cancer and the third most common cause of cancer-related death worldwide. Surgery is the best curative option for HCC treatment, but many HCC patients are not eligible for the radical treatment. Radiofrequency ablation (RFA) and transarterial chemoembolization (TACE) are local therapeutic options for unresectable HCC with encouraging local control rates. Due to recent technical advances in radiation beam delivery, radiation therapy is also considered as a locoregional treatment option for inoperable HCC [1,2].

External beam radiation therapy uses high-energy X-rays or particles to eradicate cancers. Proton beam therapy, a type of particle beam therapy, has the great advantage of precisely delivering a high radiation dose to tumour targets due to a physical property known as the “Bragg peak”, and it is now gaining prevalence worldwide as a precision radiotherapy technique [3,4]. This technique is also considered a promising modality for treating HCC because it can minimize radiation exposure to normal liver tissue and the surrounding organs, which are sensitive to radiation-induced damage [5,6,7]. Despite its superior physical dosimetry profiles, there is limited information regarding the biological impact of proton beam therapy in HCC treatment. Our recent study using 8 HCC cell lines demonstrated that relative biological effectiveness (RBE) of protons over photons varied across the cell lines [8].

Pharmacological inhibitors of histone deacetylase (HDAC), a key epigenetic regulator, were initially applied in haematological malignancies including cutaneous T cell lymphoma [9,10]. Their clinical utility has now been extended to solid cancers as a monotherapy or a combination therapy [11]. Accumulating evidence from preclinical studies indicates that radiation therapy may be a suitable combination modality with HDAC inhibitors in many types of cancers [12,13,14]. For example, suberoylanilide hydroxamic acid (SAHA; vorinostat) radiosensitizes melanoma, non-small cell lung cancer, prostate cancer, osteosarcoma and glioma [13,15,16]. DNA damage repair (DDR) genes are the major targets regulated by HDAC inhibitors and are closely related to radiosensitization [12]. However, other critical targets of HDAC inhibitors beyond DDR genes need to be investigated, especially for particle therapy.

Although proton beams are classified as low linear energy transfer (LET) radiation similar to photons, proton irradiation elicits many different cellular responses compared to X-ray irradiation [17,18,19,20]. We previously reported that epigenetic modulation via HDAC inhibitor, valproic acid, increased the sensitivity of HCC Hep3B cells to proton beams [21]. In this study, we compared the efficacy of proton radiosensitization by panobinostat (also called LBH589) in HCC cells. The radiosensitizing activity of panobinostat to X-rays has been reported in prostate cancer [22], non-small cell lung cancer [23] and bladder cancer [24], but not in HCC. In HCC, panobinostat has been tested as a monotherapy or in combination with sorafenib [25,26,27,28]. We tested the efficacy of the combination therapy in two human HCC cell lines, Huh7 and Hep3B. In addition to the previous report, our new findings further support that the combined treatment with HDAC inhibitors may be a good strategy to increase the efficacy of proton therapy in HCC.

## 2. Materials and Methods

### 2.1. Cell Culture and Drug Treatment

Two human HCC cell lines, Huh7 and Hep3B, were purchased from the Korean Cell Line Bank (Seoul, South Korea). All cell lines were cultured in Dulbecco’s Modified Eagle’s medium supplemented with 10% foetal bovine serum (Gibco, Carlsbad, CA, USA) and 1× Antibiotic-Antimycotic (Gibco). All cultures were maintained in a humidified 37 °C incubator with a 5% CO_2_ atmosphere and were routinely passaged every 2–3 days. A stock solution of panobinostat (10 μM; Selleck Chemicals, TX, USA) was made in dimethyl sulfoxide (DMSO; Sigma, St. Louis, MO, USA) and was diluted 1:1000 (10 nM) or 1:2000 (5 nM) with appropriate media. The same volume of DMSO was added to the control cell culture media as well to avoid the potential impact of DMSO on radiosensitivity. During the experiments, the media containing DMSO or panobinostat was not replaced.

### 2.2. Cell Proliferation Assay

Cell proliferation was determined using either an MTT (thiazolyl blue tetrazolium bromide) assay or a CCK-8 (Cell Counting Kit-8, Dojindo Laboratories, Kumamoto, Japan) assay. Cells were seeded at 1 × 10^3^ cells/well into a 96-well plate and were treated with various concentrations of panobinostat. Formazan formation in the viable cells was monitored by measuring the absorbance at 450 nm for the CCK-8 assays using a SpectraMax i3 microplate reader (Molecular Devices, Sunnyvale, CA, USA). The cell proliferation was calculated as a percentage relative to the untreated control.

### 2.3. Irradiation

The cells were seeded into either a 6-well plate or a 10 cm culture plate and then irradiated with either X-rays or protons the next day. The irradiation was performed using the same plates, dishes and media volumes for both X-ray and proton irradiations. For X-ray irradiation, the cell dishes were located under a 2 cm thick bolus with a source surface distance of 100 cm and a field size of 30 × 30 cm^2^, and the cells were irradiated with 6-MV photons with a dose rate of 3.96 Gy per min as previously described [29]. The photons were delivered using a Varian Clinac 6EX linear accelerator (Varian Medical Systems, Palo Alto, CA, USA). For proton beam irradiation, the cell dishes were placed at the mid-spread-out Bragg peak (SOBP), and the cells were irradiated with a 230 MeV proton beam at a dose rate of 2.14 Gy per min generated by a proton therapy system (Sumitomo Heavy Industries, Ltd., Niihama, Japan) at the Samsung Proton Therapy Center in Seoul, South Korea. The proton beam was spread out using a ridge filter to a 10 cm wide SOBP. The dosimetry profiles and verification method of the proton beam were as previously described [21].

### 2.4. Clonogenic Survival Assay

The cells were pre-treated with 5 nM panobinostat for 3 h and were irradiated with 0, 2, 4 or 6 Gy of X-rays or protons. After incubating for 21 days, the cells were fixed and stained with methanol containing 1% crystal violet, and colonies containing 50 or more cells were counted. Dose-response survival curves were compiled and analysed using GraphPad Prism, version 9.01 (GraphPad Software, La Jolla, CA, USA) as previously described [8]. Briefly, the plating efficiency was calculated as the percentage of colonies produced from the seeded cells, and cell survival at each irradiation dose was determined by dividing the plating efficiency of the irradiated cells by that of the mock-irradiated control. The RBE_37_ was defined as the ratio of X-ray doses to proton doses that reduced the fraction of surviving cells to 37% (D_37_). Sensitization enhancement ratio (SER) was defined as the ratio of D_37_ for the radiation plus DMSO to D_37_ for the radiation plus panobinostat. The plating efficiency of each experimental condition was presented in Supplementary Table S1.

### 2.5. Flow Cytometry

Apoptosis, cell cycle and reactive oxygen species (ROS) level were assessed using flow cytometry as previously described [30]. The cells were pre-treated with 5 nM panobinostat for 3 h followed by exposure to 6 Gy of X-rays or protons. For the apoptosis assay, the cells were collected at 72 h post-irradiation and stained with annexin V-FITC (BD Pharmingen, San Diego, CA, USA) and 2 μg/mL propidium iodide (PI) in annexin V binding buffer (10 mM HEPES, pH 7.4, 140 mM NaCl, 2.5 mM CaCl_2_) for 15 min at 37 °C in the dark. The apoptotic cell populations were analysed using a BD FACSVerse flow cytometer (BD Biosciences, San Jose, CA, USA). For the cell cycle analysis, the cells were collected at 24 h post-irradiation and fixed with pre-chilled 70% ethanol. The cells were incubated with 1 mg/mL RNase and 50 μg/mL PI in the dark for 30 min at 37 °C and subjected to flow cytometry. For the ROS measurement, the cells were incubated with 20 μM 2′,7′-dichlorodihydrofluorescein diacetate (DCFDA; Invitrogen, Eugene, OR, USA) for 30 min followed by exposure to 6 Gy of X-rays or protons. At 72 h post-irradiation, DCF fluorescence-based ROS levels were determined using flow cytometry and were calculated as the percentage of cells exceeding fluorescence threshold of 150 (<2% ROS levels in control cells). The data were acquired and analysed using BD FACSuite software (BD Biosciences).

### 2.6. Western Blot Analysis

Protein samples were prepared in a lysis buffer containing 20 mM Tris, pH 8.0, 137 mM NaCl, 10% glycerol, 1% nonidet P-40, 10 mM EDTA, 100 mM NaF, 1 mM phenylmethylsulfonyl fluoride, and 10 mg/mL leupeptin. Equal amounts of proteins were subjected to SDS-PAGE and transferred to nitrocellulose membranes (Bio-Rad, Richmond, CA, USA). The blots were probed with antibodies specific for the indicated proteins. The protein bands were visualized using an Amersham enhanced chemiluminescence reaction kit (GE Healthcare, Piscataway, NJ, USA) and Clarity™ Western ECL Substrate (Bio-Rad). The relative intensity of protein bands normalized to β was determined using ImageJ software (NIH, Bethesda, MD, USA). Uncropped images of all immunoblots are presented in Supplementary Figure S1.

### 2.7. siRNA Transfection

Cells (4 × 10^5^) were transfected with 10 nM siRNA duplexes designed for Mcl-1 knockdown by using Lipofectamine RNAi MAX Reagent (Thermo Fisher Scientific, Waltham, MA, USA), according to the procedure recommended by the manufacturer. Successful gene knockdown was confirmed by Western blot analysis with anti-Mcl-1 antibody. Irradiation was performed 72h after transfection. siRNAs for control (SN-1013) and Mcl-1 (#4170) were purchased from BIONEER corporation (Seoul, South Korea).

### 2.8. Statistics

The data are presented as the mean ± standard deviation (S.D.) from at least two independent experiments. The statistical analysis was performed using GraphPad Prism, version 9.01. Statistical significance was determined using one-way ANOVA.

## 3. Results

### 3.1. Panobinostat Sensitizes HCC Cells to Proton and X-ray Irradiation

**Figure 1:**
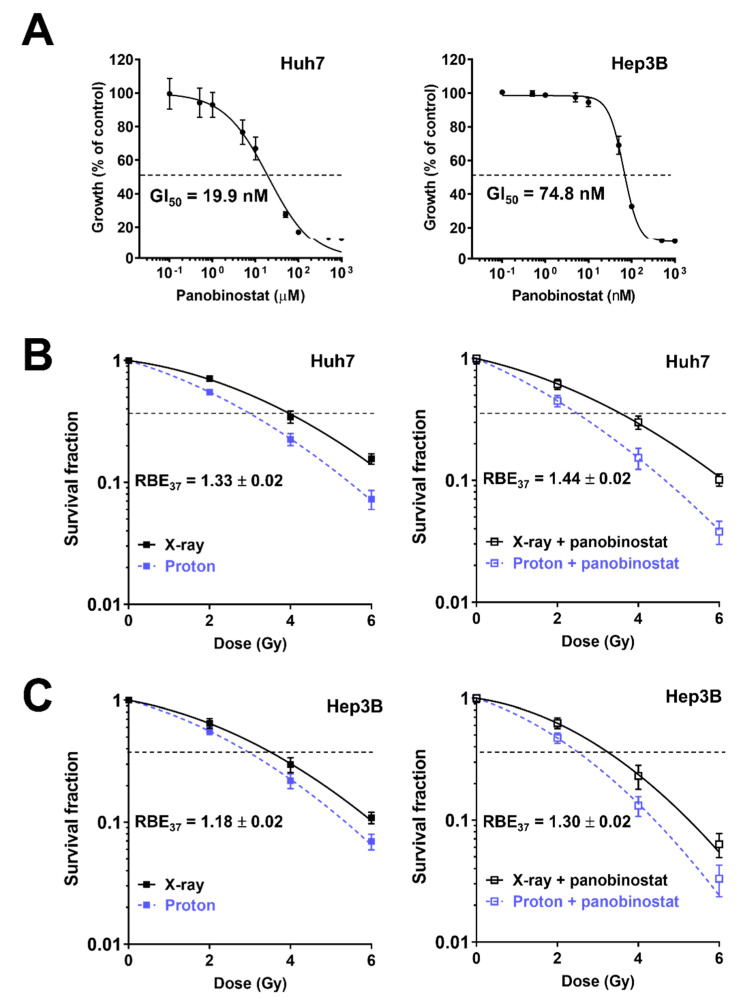
Effects of panobinostat combined with X-rays or protons on clonogenic survival of human hepatocellular carcinoma (HCC) Huh7 and Hep3B cells. (**A**) Dose-dependent inhibition of HCC cell proliferation by panobinostat. Huh7 and Hep3B cells were incubated with a range of concentrations of panobinostat for 72 h and their proliferation was determined by using the CCK-8 assay. The data represent the mean ± S.D. (n = 6). GI_50_, half maximum growth inhibition concentration. (**B**) Clonogenic survival curves show radiosensitizing activity of panobinostat to X-rays and protons in Huh7 cells. Huh7 cells were pre-treated with 5 nM panobinostat for 3 h, followed by irradiation with the indicated doses of X-rays or protons. Clonogenic assay was performed as described in Materials and Methods. Pre-incubation with panobinostat increased proton RBE. The data are expressed as the mean ± S.D. of three independent experiments performed in triplicate. (**C**) Pre-treatment with 10 nM panobinostat increased proton RBE in Hep3B cells. The data represent the mean ± S.D. of three independent experiments performed in triplicate.

We first assessed the effects of panobinostat on HCC cell proliferation using a CCK8 assay. Panobinostat inhibited the proliferation of human HCC Huh7 and Hep3B cells in a dose-dependent manner; Huh7 cells were more sensitive to panobinostat than Hep3B cells (GI_50_ = 19.9 nM versus 74.8 nM; Figure 1A). Based on the cell proliferation results, panobinostat concentration for further investigation was chosen: 5 nM for Huh7 and 10 nM for Hep3B cells, which yielded 80% to 90% cell survival. The effects of panobinostat on radiation-mediated cell killing were assessed using a clonogenic assay. The clonogenic survival curves showed Huh7 cells were more sensitive to protons than X-rays (Figure 1B). Pre-treatment with 5 nM panobinostat increased proton RBE_37_ from 1.33 to 1.44 (*p* < 0.01). Similarly, Hep3B cells had RBE_37_ of 1.18, which was significantly increased up to 1.30 by panobinostat (*p* < 0.01; Figure 1C). Comparison of SER_37_ values indicated that panobinostat sensitized Huh7 and Hep3B cells to protons to a greater extent than to X-rays (Table 1). These data indicate that panobinostat is a potent proton radiosensitizer for HCC.

### 3.2. Panobinostat Increased Sub-G1 Population When Combined with Protons in Huh7 Cells

**Figure 2:**
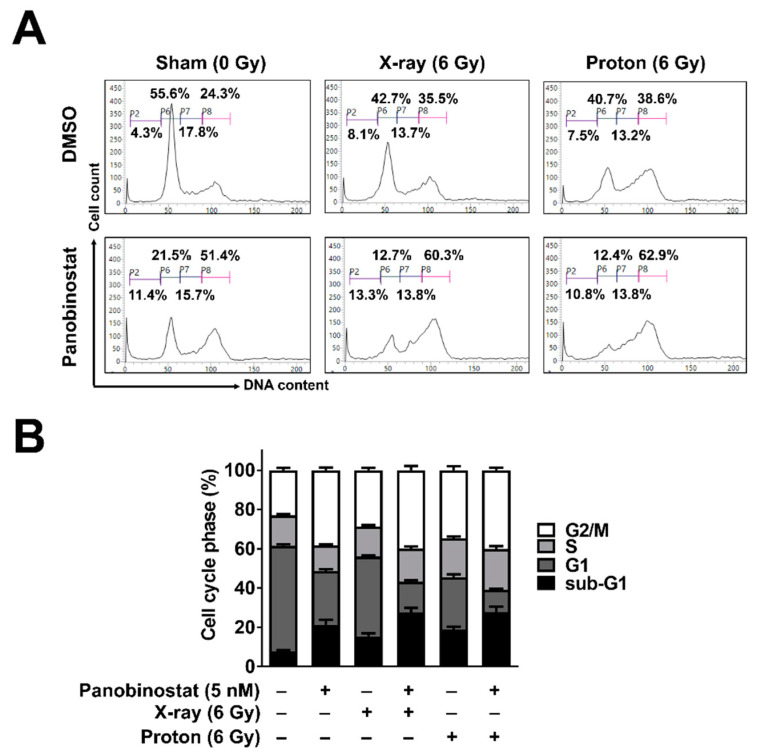
Effects of panobinostat combined with X-rays or protons on cell cycle progression in Huh7 cells. (**A**) Panobinostat induced G2/M arrest and increased the sub-G1 population when combined with radiation. Representative histograms were shown. Huh7 cells were pre-incubated with 5 nM panobinostat for 3 h and then irradiated with 6 Gy of X-rays or protons. At 24 h post-irradiation, cell cycle progression was analyzed using flow cytometry with propidium iodide staining. (**B**) Quantification of cell cycle phases. The data represent the mean ± S.D. (n = 3).

The effects of panobinostat on cell cycle progression were analysed using flow cytometry with propidium iodide staining. Panobinostat induced cell cycle arrest in the G2/M phase in Huh7 cells (Figure 2A,B); 24 h after 5 nM panobinostat treatment, the population of G2-phase cells increased from 24.3% to 51.4%. Panobinostat also increased the proportion of sub-G1 phase cells from 4.3% to 11.4%. X-ray and proton irradiation each resulted in an increase in the cell populations in the G2/M and sub-G1 phases (Figure 2A,B). When combined with panobinostat and radiation, the proportion of sub-G1 cells increased further, suggesting an enhancement of radiation-induced apoptosis by panobinostat.

### 3.3. Panobinostat Augments Proton-Induced ROS Production in Huh7 Cells

**Figure 3:**
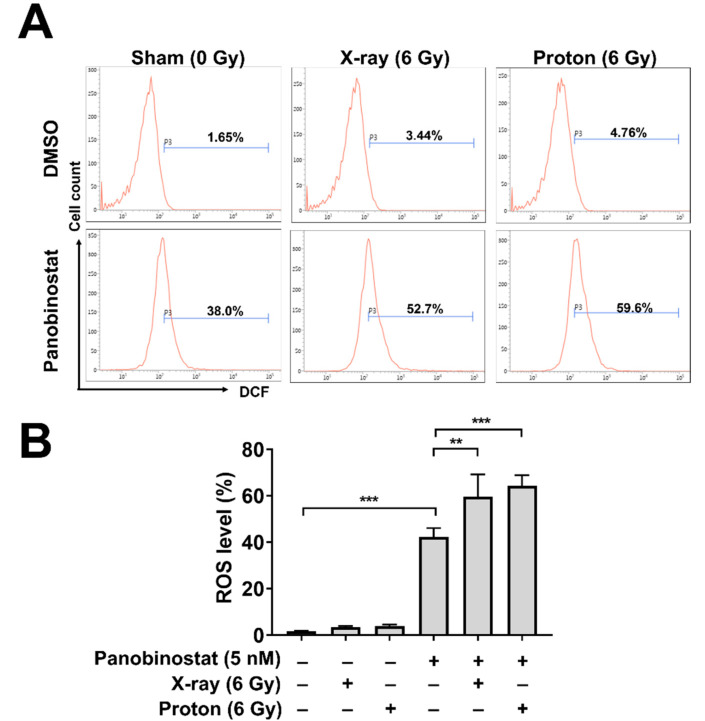
Effects of panobinostat combined with X-rays or protons on ROS generation in Huh7 cells. (**A**) Flow cytometry using DCFDA revealed that panobinostat increased ROS levels in combination with radiation. Representative histograms were shown. Huh-7 cells were pre-treated with 5 nM panobinostat and then incubated with DCFDA for 30 min, followed by irradiation with 6 Gy of X-rays or protons. Flow cytometry was performed 72 h post-irradiation. (**B**) Quantification of ROS levels in Huh7 cells. The data represent the mean ± S.D. (n = 3); ** *p* < 0.01; *** *p* < 0.001.

Ionizing radiation induces reactive oxygen species (ROS) generation mainly by two mechanisms: cellular oxidative stress and water radiolysis. For cell survival, the latter mechanism is critical due to generation of clusters of hydroxyl radicals in the vicinity of DNA. To determine the effects of panobinostat on ROS production during irradiation, we performed flow cytometry analysis using the cell-permeant ROS-sensitive dye 2′,7′-dichlorofluorescin diacetate (DCFDA). Panobinostat alone increased the ROS level from 1.65% to 38.0% (*p* < 0.001; Figure 3A,B). Increased ROS production was also seen in Huh7 cells after irradiation with 6 Gy of either X-rays (from 1.65 to 3.44%) or protons (from 1.65 to 4.76%) (Figure 3A,B). Pre-treatment with 5 nM panobinostat further augmented ROS production when combined with X-ray (52.7%) or proton irradiation (59.8%).

### 3.4. Panobinostat Enhances Proton-Induced DNA Damage in Huh7 Cells

**Figure 4:**
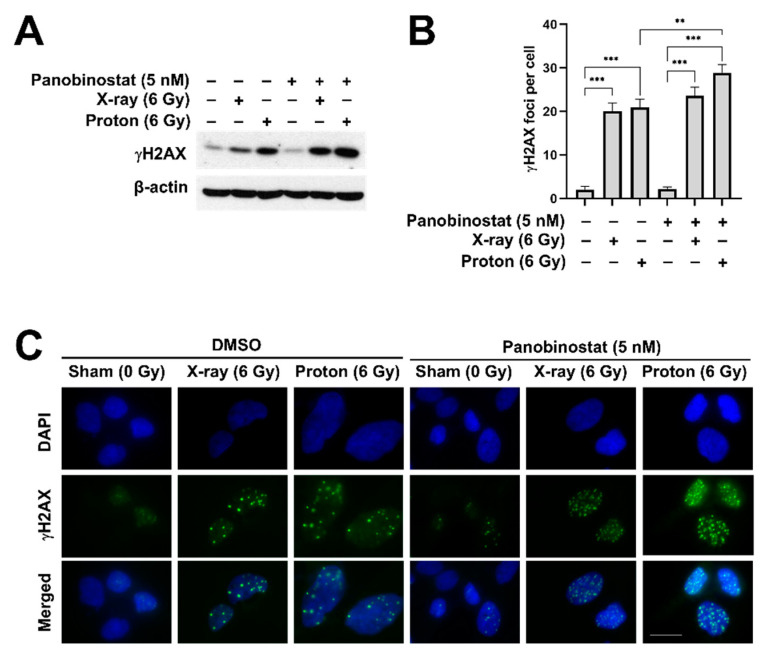
Effects of panobinostat combined with X-rays or protons on DNA damage in Huh7 cells. Huh7 cells were plated on cover slips and then were treated with 5 nM panobinostat 3 h prior to irradiation. The cells were irradiated with 6 Gy of X-rays or protons and the samples were prepared 24 h post-irradiation. (**A**) Western blotting showed that expression of γH2AX, a sensitive marker for DNA double-stranded breaks, was increased by either X-rays or protons, which was further enhanced by pre-treatment with panobinostat. β-actin was used as a loading control. (**B**) Quantification of γH2AX foci in the nuclei of the Huh7 cells irradiated with X-rays or protons. The data represent the mean ± S.D. (n = 3); ** *p* < 0.01; *** *p* < 0.001. (**C**) Representative immunofluorescence images of γH2AX. Huh7 cells were pre-treated with 5 nM panobinostat, followed by 6 Gy of X-rays or protons. The cells were fixed and stained 24 h post-irradiation. Blue: DAPI; green: γH2AX; scale bar: 20 μm.

To test whether panobinostat affects radiation-induced DNA damage, we determined the expression of γH2AX, a surrogate marker for DNA double strand breaks (DSBs), in Huh7 cells after X-ray or proton irradiation. Western blot analysis showed that the expression of γH2AX increased 24 h post-irradiation, which was further enhanced by 5 nM panobinostat (Figure 4A). Immunofluorescence staining of γH2AX showed that either X-rays or protons increased the number of γH2AX foci in the nucleus (Figure 4B,C). Panobinostat significantly increased proton-induced formation of γH2AX foci (*p* < 0.01; Figure 4B). Panobinostat alone did not induce DSBs, as evidenced by both western blot and immunofluorescence.

### 3.5. Panobinostat Enhances Proton-Induced Apoptosis Possibly through Downregulation of Mcl-1 Expression in HCC Cells

**Figure 5:**
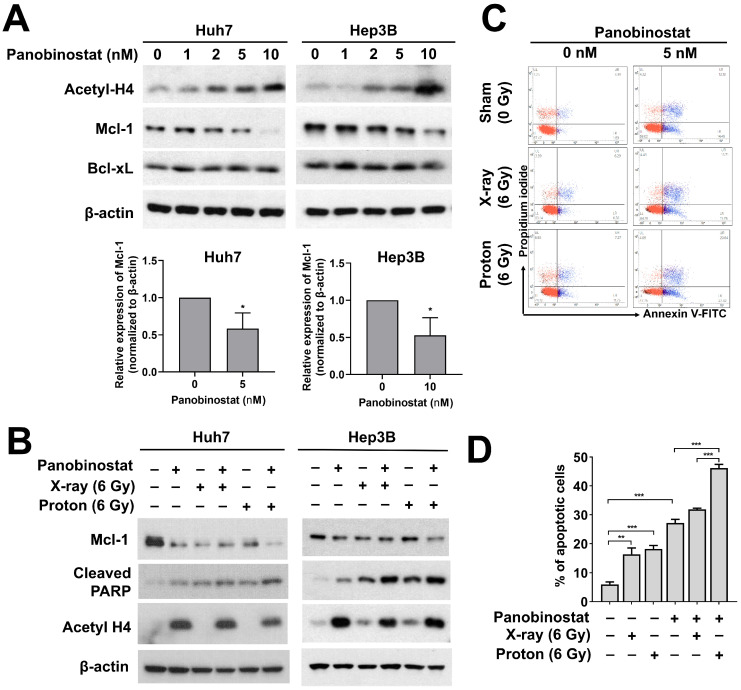
Effects of panobinostat combined with X-rays or protons on apoptotic signaling in HCC cells. (**A**) Huh7 and Hep3B cells were treated with the indicated concentrations of panobinostat and were harvested at 24 h. Western blot analysis showed that panobinostat decreased Mcl-1 but not Bcl-xL in a concentration-dependent manner. Concomitant enrichment of acetylated histones H4 was also seen. β-actin was used as a loading control. Relative expressions of Mcl-1 normalized to β-actin were presented. The data represent the mean ± S.D. (n = 3); * *p* < 0.05. (**B**) Co-treatment with panobinostat and radiations further induced apoptotic cell death in HCC cells. HCC cells were pre-treated with panobinostat (5 nM for Huh7 and 10 nM for Hep3B) for 3 h, followed by 6 Gy of X-rays or protons. The cells were harvested 72 h post-irradiation. The combination with panobinostat and proton further decreased Mcl-1 expression with an increase in cleaved PARP expression, a surrogate marker for apoptosis. β-actin was used as a loading control. (**C**) Flow cytometry showed enhanced apoptotic cell death in response to co-treatment with panobinostat and proton irradiation in Huh7 cells. Apoptosis was detected by Annexin V/propidium iodide double staining. Representative flow cytometry scatter plots were shown. (**D**) Quantification of apoptotic cell population. The data represent the mean ± S.D. (n = 3); ** *p* < 0.01; *** *p* < 0.001.

Next, we investigated the effects of panobinostat on the expression of anti-apoptosis-related proteins such as anti-apoptotic Mcl-1and Bcl-xL in HCC cells. Panobinostat dose-dependently increased the acetylation of histones H4 in Huh7 and Hep3B cells (Figure 5A). Panobinostat decreased the expression of Mcl-1 at 24 h in a dose-dependent fashion. At 10 nM of panobinostat, Mcl-1 level was almost absent and cleaved PARP level was strongly induced (Figure 5A).

Based on the stimulatory effect of panobinostat on apoptosis, we tested effects of panobinostat combined with either X-ray or proton irradiation on apoptosis in Huh7 and Hep3B cells. Pre-treatment with 5 nM panobinostat decreased Mcl-1, which was further suppressed by co-treatment with proton irradiation (Figure 5B). Both HCC cells had higher expression of cleaved PARP in combination with panobinostat and radiation compared to radiation or panobinostat alone (Figure 5B). The enhanced apoptosis due to co-treatment was further confirmed using flow cytometry with annexin V/propidium iodide double staining (Figure 5C,D). Irradiation with X-rays and protons increased the population of apoptotic cells from 5.99% to 16.3% and 18.2%, respectively. Panobinostat alone induced apoptosis (27.2%), and it greatly enhanced apoptosis in combination with protons (46.2%) to a greater extent than X-rays (31.9%; *p* < 0.001). These data suggest the enhanced apoptosis through suppression of Mcl-1 may lead to panobinostat-mediated proton sensitization in HCC cells.

### 3.6. Depletion of Mcl-1 Increases Proton Sensitivity in Huh7 Cells

**Figure 6:**
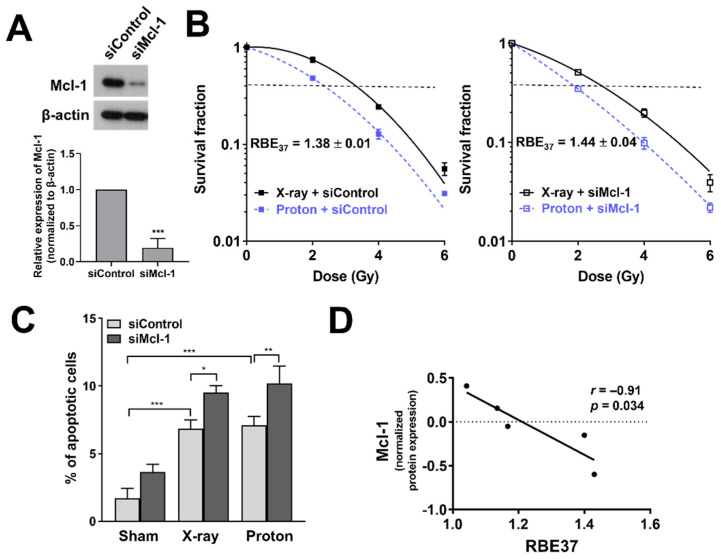
Effects of Mcl-1 depletion on proton radiosensitization in Huh7 cells. (**A**) siRNA-mediated knockdown of Mcl-1 in Huh7 cells. Western blot confirmed reduction of Mcl-1 protein level. β-actin was used as a loading control. Relative expressions of Mcl-1 normalized to β-actin were presented. The data represent the mean ± S.D. (n = 3); *** *p* < 0.001. (**B**) Clonogenic survival curves showed that proton RBE was increased by Mcl-1 knockdown. The data represent the mean ± S.D. of three independent experiments performed in triplicate. (**C**) Flow cytometry showed that the depletion of Mcl-1 enhanced proton-induced apoptosis. The data represent the mean ± S.D. (n = 3); * *p* < 0.05; ** *p* < 0.01; *** *p* < 0.001. (**D**) Correlation analysis between Mcl-1 level and RBE_37_ of HCC cell lines. The normalized protein expression data and RBE values of five HCC cell lines, Huh7, Hep3B, HepG2, SK-HEP-1 and SNU449 was used for the analysis. Pearson correlation coefficient (r) and *p*-value are shown.

Given that Mcl-1 may be related to proton sensitivity, we investigated the effect of Mcl-1 depletion on proton radiosensitization in Huh7 cells. Western blot analysis showed that Mcl-1 was depleted by siRNA treatment in Huh7 cells (Figure 6A). Clonogenic assay with control siRNA-treated Huh7 cells showed that proton irradiation decreased clonogenic survival to a greater extent than X-ray irradiation (Figure 6B). Mcl-1 depleted cells were more sensitive to protons than X-rays, as evaluated by SER_37_ (1.31 versus 1.26, not significant; Table 2). The Mcl-1 siRNA treatment increased proton RBE_37_ from 1.38 to 1.44 (*p* < 0.05; Table 2 and Figure 6B). Apoptosis assay using flow cytometer showed that Mcl-1 siRNA treatment increased the percentage of apoptotic cells and further enhanced it when combined with X-rays (*p* < 0.05) or protons (*p* < 0.01), compared to control siRNA treatment (Figure 6C). However, no significant difference in apoptosis of Mcl-1-depleted cells between X-rays and proton irradiations was observed. To further confirm whether Mcl-1 level affects RBE, we reanalyzed proton RBE_37_ values used for our previous report [28] and took normalized expression data of Mcl-1 from a previous quantitative proteomics study of 375 cancer cell lines [29]. We selected five HCC cell lines, Huh7, Hep3B, HepG2, SK-HEP-1 and SNU449 because they have both Mcl-1 protein level and proton RBE data. Correlation analysis revealed a strong negative correlation between Mcl-1 and RBE*_37_* (*p* < 0.05; Figure 6D).

## 4. Discussion

Proton beam therapy is one of the most advanced technologies for cancer treatment [3,4]. While the physics of proton beam therapy in cancer treatment are well characterized, far less is understood about its biology. A comparison of the biological consequences between proton and X-ray irradiation, called the proton relative biological effectiveness (RBE), suggests that a proton beam is approximately 10% more effective than X-rays, which is taken into account in clinical practice with a fixed RBE value of 1.1 [31]. However, more comprehensive studies on protons’ biological effects suggest that this assumption is too simplistic and that proton irradiation differentially modulates diverse signalling pathways, such as apoptosis, cell cycle and angiogenesis, which are critical to radiation treatment response [17,18,19,20]. In recent years, much attention has been paid to the role of epigenetic regulation by diverse histone modifications during radiation therapy as previously reviewed [12]. Our recent study showed enhancement of in vitro and in vivo radiosensitization by valproic acid in HCC Hep3B cells [21], but its clinical use may be limited because of hepatotoxicity issue [32]. In this study, we compared the efficacy of another potent HDAC inhibitor, panobinostat in sensitization of HCC cells to proton irradiation.

Since deregulated epigenetic modification through aberrant HDAC expression results in carcinogenesis, numerous HDAC inhibitors have been developed and clinically tested [9,10,11]. Panobinostat suppresses HCC cell proliferation via inhibition of DNA methyltransferase activity [26] or inhibition of the gankyrin/STAT3/Akt pathway [27]. Autophagy-related cell death is another panobinostat’s anti-cancer mechanisms in HCC cells [33]. Our data showed that panobinostat induced cell cycle arrest at the G2 phase in HCC cells, possibly through downregulating Chk1 expression as previously shown in non-small cell lung cancer cells [34]. In addition, panobinostat augmented ROS production in HCC cells, which is consistent with a previous study in cervical cancer cells [35]. It is therefore likely that panobinostat-mediated Chk1 downregulation and ROS accumulation would make HCC cells more vulnerable to irradiation. Our recent study using triple negative breast cancer cells supports the notion that Chk1 inhibition enhances proton sensitivity [36]. Although panobinostat alone increased ROS level from 1.65% to 38.0%, this increase is not associated with cell clonogenic death because a marginal increase in ROS level by X-rays from 1.65% to 3.44% led to approximately 90% cell death, thus indicating no correlation between ROS level and clonogenic death.

In addition to conventional X-rays, particle therapy using protons or carbon ions has been recently tested in combination with HDAC inhibitors [21,37,38,39,40,41]. Our previous study showed that valproic acid (VPA), an antiepileptic drug with HDAC-inhibition activity, enhanced proton radiosensitization of HCC cells in vitro and in vivo [21]. Enhanced accumulation of ROS after proton irradiation leads to activation of the key redox transcription factor NRF2, which is suppressed by VPA resulting in proton sensitization [21]. Vorinostat enhances apoptosis and suppresses clonogenic survival by inducing G1 phase arrest and has a greater synergistic effect in melanoma cells when combined with carbon ions than with X-rays [41]. Vorinostat also sensitizes diverse cancer cells, including glioma, sarcoma and lung cancer, to carbon ion irradiation [37,38,40].

Our data further revealed a previously unrecognized role of Mcl-1 in proton radiosensitization. Loss of function study showed that Mcl-1 depletion resulted in the enhancement of proton radiosensitization and RBE_37_ (Figure 6). This suggests that panobinostat-induced proton sensitization may be partly caused by loss of Mcl-1 in Huh7 cells. We cannot rule out the possibility that Mcl-1 did not decrease for 3 h after panobinostat treatment, which means that the Mcl-1 levels were different between panobinostat-treated cells and Mcl-1 knockdown cells. Nonetheless, the results of the two experiments appear similar, suggesting that Mcl-1 may be more important to post-irradiation recovery process such as DDR repair than at the time of irradiation. Mcl-1 is a target protein of Specificity protein 1 (Sp1) and panobinostat downregulates via Sp1 suppression in oral squamous cell carcinoma [42]. Panobinostat resistance of cutaneous T-cell lymphoma (CTCL) cells is related to high Bcl2 family expression; Bcl-2 inhibitor sensitized CTCL cells to panobinostat [43]. In addition to a well-established role in the mitochondrial apoptosis, Mcl-1 regulates DNA damage repair (DDR) in response to nuclear DNA damage stress such as ionizing radiation. Mattoo et al. demonstrated that Mcl-1 depletion impairs homologous recombination (HR) repair pathway [44]. Growing evidence indicates that cells with defects in HR-mediated DDR pathway are more sensitive to proton irradiation than X-ray [45,46]. Thus, we speculate that panobinostat-induced downregulation of Mcl-1 may impede HR pathway, leading to an increase in proton RBE. The negative correlation between Mcl-1 level and proton RBE (Figure 6D) could support this hypothesis, even though the sample size may be too small. Further studies are warranted to fully elucidate the association between Mcl-1 and proton RBE and the underlying biological mechanism.

Since the Food and Drug Administration approved panobinostat for multiple myeloma in 2015, panobinostat has been clinically tested for many types of cancers as a monotherapy or a combination therapy. For HCC treatment, sorafenib was the only combination option to test the synergism with panobinostat [25]. Our current study suggests the strong sensitizing effect of panobinostat to both X-rays and protons in HCC cell lines. Proton beam radiation, which is being actively used for HCC management in the clinic, may be a particularly promising combination strategy with panobinostat for unresectable HCC patients.

## 5. Conclusions

Our findings propose another epigenetic drug, panobinostat, as a proton radiosensitizer for HCC treatment. Downregulation of Mcl-1 by panobinostat may be one of the plausible mechanisms for proton radiosensitization. This study provides evidence that proton beam therapy could be improved by modulation of anti-apoptotic proteins related to chemo-and radio-resistance.

## Figures and Tables

**Table 1:** Radiation response parameters of panobinostat-treated Huh7 and Hep3B cells.

Cell Line/Treatment	D_37_ (Gy)	SER_37_ (DMSO Versus Panobinostat)	RBE_37_ (X-ray Versus Proton)
Huh7			
X-ray, DMSO	3.96 ± 0.21		
X-ray, panobinostat	3.45 ± 0.30	1.15 ± 0.04	
Proton, DMSO	2.99 ± 0.12		1.33 ± 0.02
Proton, panobinostat	2.41 ± 0.25	1.25 ± 0.08	1.44 ± 0.02 **
Hep3B			
X-ray, DMSO	3.54 ± 0.30		
X-ray, panobinostat	3.20 ± 0.32	1.11 ± 0.02	
Proton, DMSO	3.00 ± 0.21		1.18 ± 0.02
Proton, panobinostat	2.47 ± 0.21	1.21 ± 0.02 **	1.30 ± 0.02 **

Data are mean ± S.D. The *p*-values were calculated by unpaired two-tailed Student’s t test. ** *p* < 0.01. D_37_, radiation dose at 37% cell survival; SER, sensitization enhancement ratio; RBE, relative biological effectiveness.

**Table 2:** Radiation response parameters of Mcl-1 depleted Huh7 cells.

Cell Line/Treatment	D_37_ (Gy)	SER_37_ (siControl versus siMcl-1)	RBE_37_ (X-ray versus Proton)
Huh7			
X-ray, siControl	3.44 ± 0.06		
X-ray, siMcl-1	2.74 ± 0.04	1.26 ± 0.02	
Proton, siControl	2.50 ± 0.04		1.38 ± 0.01
Proton, siMcl-1	1.90 ± 0.05	1.31 ± 0.04	1.44 ± 0.04 *

Data are mean ± S.D. The *p*-values were calculated by unpaired two-tailed Student’s *t* test. * *p* < 0.05. D_37_, radiation dose at 37% cell survival; SER, sensitization enhancement ratio; RBE, relative biological effectiveness.

## Data Availability

All the data presented in this study are included in this article and its Supplementary Materials File.

## Supplementary Materials

The following are available online at https://www.mdpi.com/2073-4409/10/3/554/s1, Figure S1: Uncropped images of western blots. Table S1: Plating efficiency of Huh7 and Hep3B cells treated with DMSO, panobinostat and siRNAs.
