# Supplementary material for: Downregulation of Mcl-1 by Panobinostat Potentiates Proton Beam Therapy in Hepatocellular Carcinoma Cells
Source: Cells. 2021 Mar 4;10(3):554. doi: 10.3390/cells10030554 (PMC7999709; doi:10.3390/cells10030554)
Supplement: Supplementary file 1 [file cells-10-00554-s001.pdf]

Fig. 4A

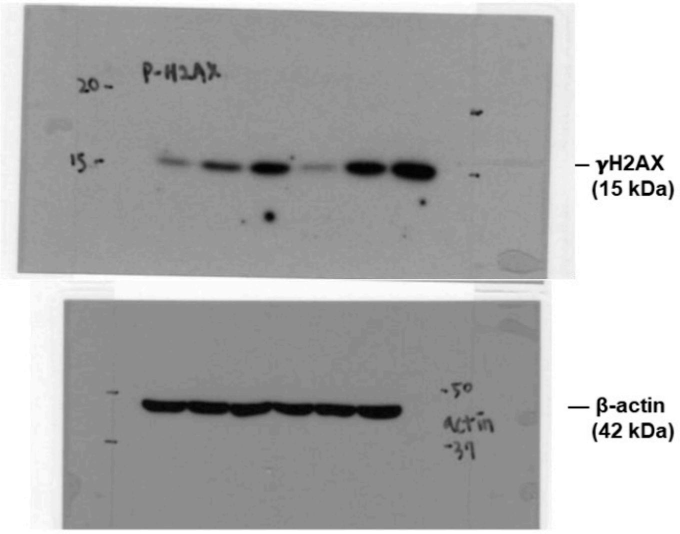

Fig. 5A

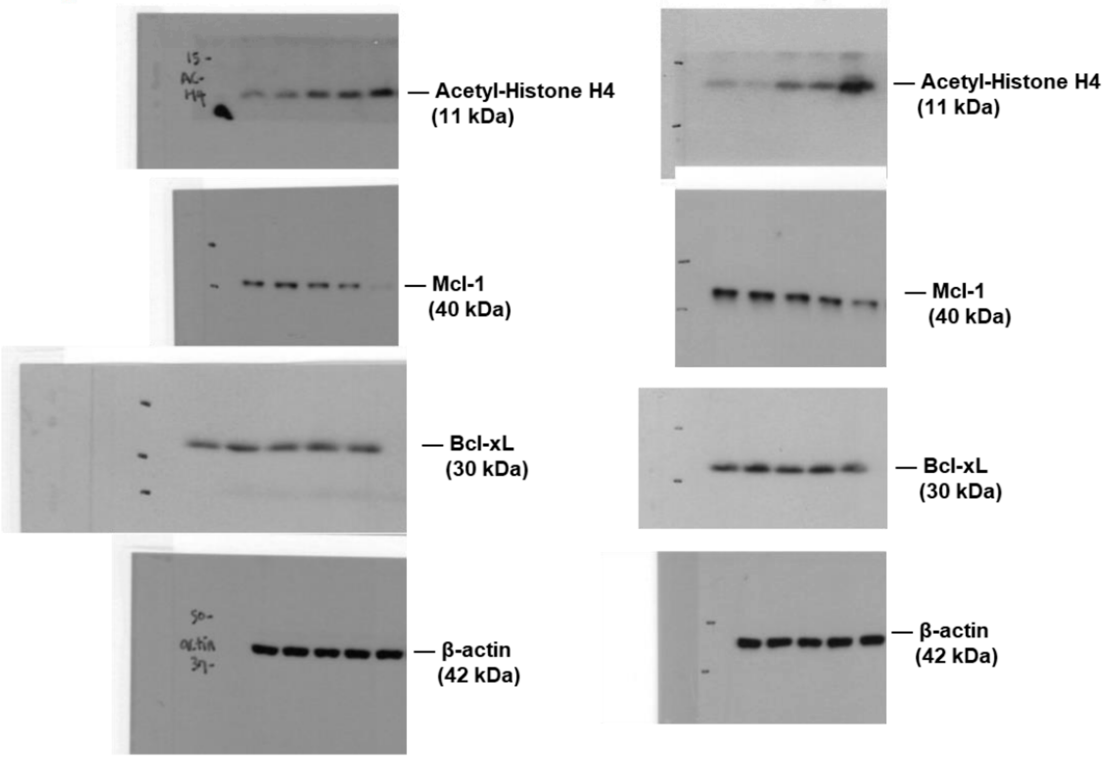

Supplementary Figure S1. Uncropped images of western blots.

Fig. 5B

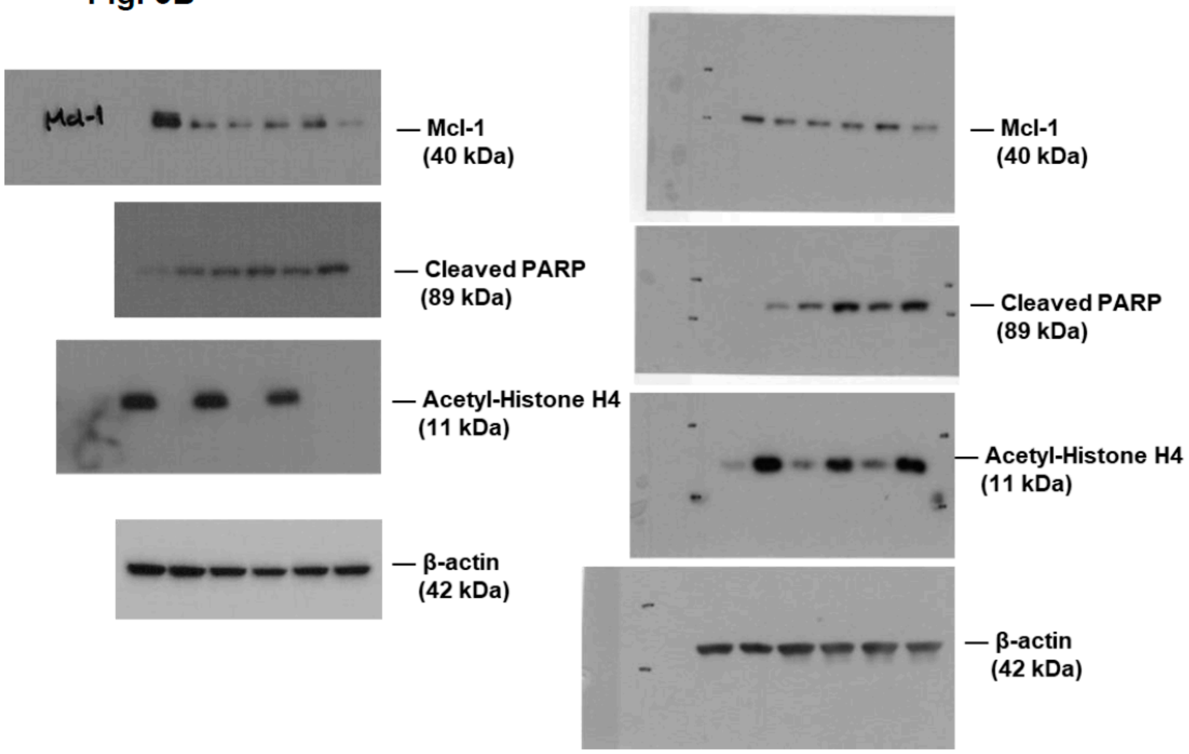

Fig. 6A

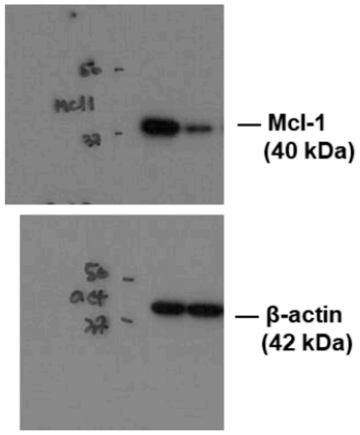

Supplementary Figure S1. Uncropped images of western blots (continued).

Supplementary Table S1. Plating efficiency of Huh7 and Hep3B cells treated with DMSO, panobinostat and siRNAs.

Cell line	Treatment	Plating efficiency (%)
Huh7	DMSO	32.44 ± 3.69
	Panobinostat	27.67 ± 2.31
Hep3B	DMSO	46.11 ± 2.34
	Panobinostat	39.33 ± 1.67
Huh7	siControl	32.33 ± 4.31
	siMcl-1	24.17 ± 3.55
